# Lessons learnt from a nationally funded training and mentoring programme for early‐mid career musculoskeletal researchers in Australia

**DOI:** 10.1002/msc.1820

**Published:** 2023-10-16

**Authors:** Belinda J. Lawford, Rana S. Hinman, Kim L. Bennell, David J. Hunter, Paul W. Hodges, Jenny Setchell, Jillian Eyles, Kim Allison, Penny Campbell, Rebecca Mellor, MUSQ:Impact Steering Committee, Laura Diamond, Kade Paterson, Stephanie Filbay, Megan Ross, B. Vicenzino

**Affiliations:** ^1^ Centre for Health, Exercise and Sports Medicine Department of Physiotherapy School of Health Sciences The University of Melbourne Melbourne Victoria Australia; ^2^ Sydney Musculoskeletal Health Arabanoo Precinct Kolling Institute Faculty of Medicine and Health The University of Sydney Sydney New South Wales Australia; ^3^ The University of Queensland Centre of Research Excellence in Spinal Pain Injury and Health School of Health and Rehabilitation Sciences Brisbane Queensland Australia

**Keywords:** early career researchers, mentoring, musculoskeletal, training

## Abstract

**Introduction:**

Quality training and mentoring are crucial components of successful career development for early mid career researchers (EMCRs). This paper describes the overarching framework of novel ongoing national Training and Mentoring Programme Melbourne University Sydney Queensland:Impact (MUSQ:Impact) for musculoskeletal researchers, including a description of how it was set up and established, and lessons learned from its implementation.

**Results:**

The MUSQ:Impact programme spans four multidisciplinary musculoskeletal research teams across three universities in Australia, comprising 40–60 EMCR members. It was established to provide EMCRs with a unique learning environment and opportunities to gain exposure to, and network with, other national musculoskeletal research teams. Specific goals are to focus on core research competencies (e.g. writing skills, managing grant budgets, public speaking and media engagement, research translation), provide career mentoring, fund development activities (e.g. conference attendance, laboratory visits, skill development courses), and share training resources (e.g. data dictionaries, project summaries). A Steering Committee of 10–12 EMCR members, co‐chaired by a senior researcher and one EMCR, is responsible for overseeing MUSQ:Impact and organising regular activities, including a monthly webinar series, a mentor/mentee scheme, annual group research retreats, annual infographic competition, and funding awards. An evaluation survey found that most participants perceived each activity to be beneficial and of value to their research career and development.

**Conclusion:**

This paper presents the structure of national training and mentoring programme that serves as a potential template for other research teams to adapt within their own contexts.

## 1 INTRODUCTION

Quality training and mentoring are crucial components of successful career development for health scientists (Sambunjak et al., 2006). It has been identified as an important stimulus to enhance the research success of graduate and early mid career researchers (EMCRs) (Diggs‐Andrews et al., 2021; Merga & Mason, 2021; Schriever & Grainger, 2019) and retention of clinician‐scientists in research (Kupfer et al., 2002). Positive training and mentoring experiences have been linked to greater productivity, career satisfaction, and research success of mentees (Beech et al., 2013; Bland et al., 2009; Cho et al., 2011; Feldman et al., 2010; Fleming et al., 2012; McGee & Keller, 2007; Sambunjak et al., 2010; Shea et al., 2011). For mentors, the opportunity to ‘give back’ through supporting EMCRs can contribute to overall work satisfaction (Charron et al., 2019). The lack of quality training and mentoring has been identified as a major factor hindering career progress in academic health sciences (Jackson et al., 2003).

Within research institutions, the success of clinical translational research programs depends on quality training and mentoring to foster the career development of EMCRs. Successful research career development is dependent not only on robust training and mentoring programs but also on opportunities for networking, cross‐disciplinary research, and collaboration (Bakken et al., 2006). To our knowledge, there are few published detailed descriptions of formalised training and mentoring programs for EMCRs in health science musculoskeletal research (Johnson et al., 2010; Pfund et al., 2014; Pfund et al., 2006; Allen et al., 2006a). Doing so has the potential to help guide others who are interested in establishing their own programme.

This paper describes a novel ongoing Training and Mentoring Programme initiated with funding from Australian National Health and Medical Research Council (NHMRC) grants and involving four musculoskeletal research teams across three institutions, the Universities of Melbourne, Sydney, and Queensland—known as ‘Melbourne University Sydney Queensland:Impact’ (or MUSQ:Impact). MUSQ:Impact aims to provide research training and mentoring to EMCRs to foster successful career development. This manuscript describes the overarching structure of MUSQ:Impact, how it was set up and established, and lessons learned from its implementation, with the aim of enabling other teams to develop programs within their own research teams and contexts.

## 2 GOALS OF THE MUSQ:IMPACT PROGRAMME

**FIGURE 1:**
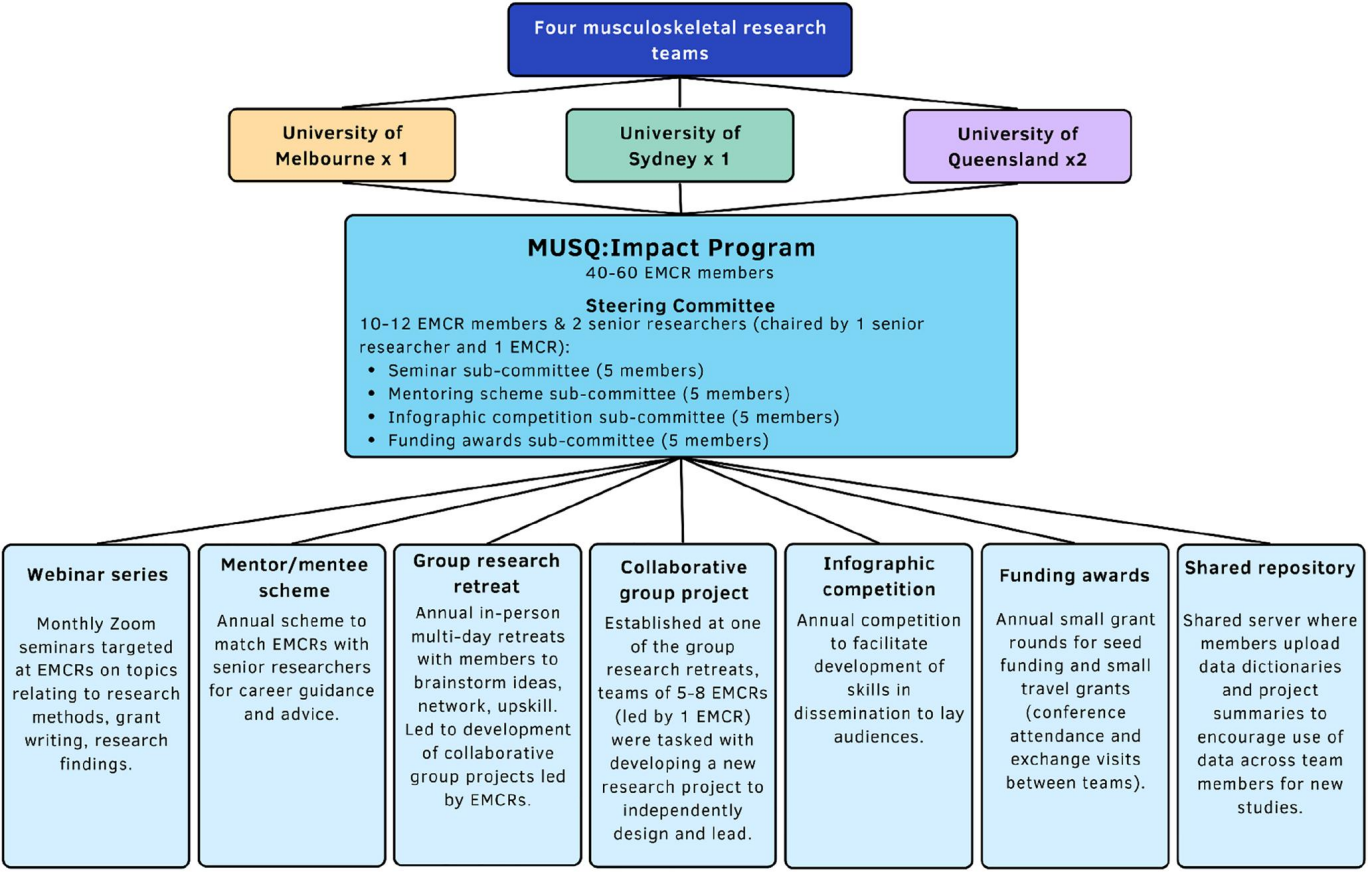
Overview of the MUSQ:Impact training and mentoring programme.

The MUSQ:Impact Programme (Figure 1) was established in 2015 by four senior musculoskeletal researchers from three different Australian states (University of Melbourne, University of Sydney, and University of Queensland) following funding via an Australian National Health and Research Council (NHMRC) Centre of Research Excellence grant (APP1079078) and a NHMRC Programme grant (#1091302) in musculoskeletal disorders. These senior researchers lead their own multi‐disciplinary musculoskeletal research teams within each site, ranging from translational research and clinical trials to biomechanics and basic science research. Each group comprises approximately 10–20 EMCRs (including graduate research students). All EMCRs and professional staff within each research team are invited to participate in MUSQ:Impact, resulting in a total of 50–60 members at any one time.

MUSQ:Impact was established to provide a unique learning environment for EMCRs and opportunities to gain exposure to other national musculoskeletal research teams. Specific goals are to:

- Focus on core research competencies—including skills in writing scientific/lay publications, preparation of grant applications and budgets, undertaking media interviews, presenting to clinical, scientific and lay audiences, reviewing journal articles and grants, and developing research translation skills;
- Enable regular participation in cross‐institution activities such as monthly research webinars/research retreats to provide exposure to a range of research projects under the Programme's umbrella, as well as provide opportunities to socialise and consult with other members of the teams;
- Provide career mentoring for optimisation of research careers;
- Fund development activities (e.g. conference participation, laboratory visits, and skill development courses);
- Share training resources (e.g. data dictionaries, project summaries) between sites via a dedicated computer server;
- Provide leadership opportunities for EMCRs.

## 3 GOVERNANCE STRUCTURE

At the inception of MUSQ:Impact, the senior researchers wrote detailed terms of reference and established a Steering Committee of 10–12 members made up of two senior researchers and EMCRs from each of the four teams ‐ co‐chaired by one senior researcher and one EMCR. Each EMCR Committee member serves a 2‐year term before a new member is recruited from the participating teams. The Committee leads the organisation of numerous activities/initiatives that aim to foster successful career development of participants, provide opportunities for cross‐disciplinary and cross‐institution networking and collaboration, and provide mentoring support for EMCRs. These activities align with recommendations and best practices for mentoring and career development programs (Allen, 2009; Elements of Effective Practice for Mentoring[TM], 2015; Lacerenza et al., 2017) and include a monthly seminar series, a mentee/mentor scheme, group research retreats, collaborative research projects, infographic competitions, seed funding awards and travel grants. The terms of reference are reviewed periodically by the Committee and senior researchers to ensure they remain appropriate based on learnings from the previous activities.

The Committee meets quarterly via Zoom and is responsible for organising all MUSQ:Impact activities/initiatives and reporting back to the senior researchers. Committee members form dedicated sub‐committees (of approximately 5–8 EMCR members) who are responsible for the organisation of webinars, the mentoring programme, research retreats, an infographic competition, and funding awards (for inter‐institutional exchanges, to support small EMCRs research projects, and to support conference attendance). The Committee is allocated funds annually to dedicate to the MUSQ:Impact activities/initiatives.

## 4 ACTIVITIES AND INITIATIVES

A number of dedicated MUSQ:Impact activities and initiatives are conducted annually. The key activities/initiatives are described here.

### 4.1 Webinar series

Zoom webinars are held monthly and all programme members from each of the four teams are invited to attend. The overarching aim of the series is to build EMCR research skills and capabilities. Seminar topics are informed by preference (ascertained via annual surveys to MUSQ:Impact members), and include topics on specific research methodologies, grant writing skills, and findings from relevant studies. Guest speakers are invited either from within or outside the MUSQ:Impact institutions. All seminars are coordinated and organised by a seminar sub‐committee, led by an EMCR.

### 4.2 Mentor/mentee scheme

Throughout the MUSQ:Impact programme, two rounds of a mentor/mentee matching scheme have been held, organised by a dedicated sub‐committee and led by an EMCR. The purpose of this scheme is to utilise the experience of more senior researchers within MUSQ:Impact to support the development of EMCR skills required for a successful research career. This scheme also provides mid‐career researchers with an opportunity to improve their mentoring skills by mentoring junior staff/students. The scheme is open to all senior researchers, students, EMCRs, and professional staff and operates on an opt‐in basis, whereby staff nominate themselves to be involved either as a mentor or a mentee. As per recommendations for career mentoring programs (Lumpkin, 2011), mentees are matched with a more senior mentor, ideally from a different institution/team to their own. Each iteration of the mentee/mentor scheme lasts for 1 year. The scheme is unstructured, whereby it is up to the mentee‐mentor pair to arrange meetings as required and determine discussion topics.

### 4.3 Group research retreats

Group research retreats are held on a semi‐annual basis and attended by approximately 20–25 MUSQ:Impact members (typically EMCRs and the four senior researchers). This includes a mix of attendees from each of the four teams as well as invited expert guests/speakers (including international guests) who come together in‐person over a 3–4 day period. Retreats are fully funded by the senior researcher's grants and provide an opportunity for MUSQ:Impact members to discuss/brainstorm collaborative group projects, network, and learn new skills (e.g. workshops on generating research impact). Retreats have been organised by Committee members who formed the retreat organising sub‐committee. Each retreat has a unique purpose/theme. The retreats held to date include:

1. 2016 retreat in Queensland: ‘How do we identify sub‐groups of people with musculoskeletal disorders who respond to different treatments?’
2. 2017 retreat in New South Wales: ‘Mechanisms underlying development, maintenance, and progression of musculoskeletal disorders: Collaborative project’.
3. 2018 retreat in Queensland: ‘Conducting impactful research’
4. 2020 virtual retreat (via Zoom): ‘Mine the gap—linking basic science and clinical research’ (Duong et al., 2021)
5. 2022 retreat in Queensland: ‘Influencing policy, the use of social media, and big data in musculoskeletal research—collaborative research projects’

### 4.4 Collaborative projects

Collaborative group projects have been established at two of the MUSQ:Impact research retreats (2017 and 2022), where three teams of 5–8 EMCRs are tasked with developing a new research project to independently design and lead. The aim of these collaborative projects is to lead to at least one journal publication, encourage collaboration and teamwork across MUSQ:Impact sites, and foster the development of independent leadership skills. Each collaborative project is led by an EMCR, who is responsible for driving the project, organising team meetings with other EMCR members, and delegating tasks. Projects were conceived at the research retreat and continued over the next 1–2 years, with new EMCRs able to join the team at any timepoint.

### 4.5 Infographic competitions

Throughout the MUSQ:Impact programme, three rounds of infographic competitions have been held. These competitions intend to facilitate the development of skills in research dissemination to lay audiences. All MUSQ:Impact EMCRs across the three sites were invited to participate. Small monetary prizes are awarded for overall winners ($800 AUD) and runners up ($300 AUD each). Winning infographics are disseminated via social media. In 2020, all MUSQ:Impact members were invited to attend a full‐day data visualisation course (funded by the senior researcher's grants) to learn how to create and design research infographics.

### 4.6 Funding awards

Grant funding rounds are held during the MUSQ:Impact programme to assist EMCRs to develop their track records and build skills relevant to a research career. This has included a Translational Project Grant (up to $25,000 AUD) to provide seed funding for pilot data that might lead to larger projects/funding. Small grants (up to $1000 AUD for individuals and $4000 for groups) are also awarded to support graduate researchers and EMCRs to travel and spend time with another MUSQ:Impact site or for a group to lead a workshop/training activity. All EMCRs from each site were invited to submit applications.

## 5 EVALUATION OF THE MUSQ:IMPACT PROGRAMME

An important component of the MUSQ:Impact programme was to conduct an evaluation of user perceptions and experiences to determine what elements were, or were not, working. In the fifth year of MUSQ:Impact, all members (including those who had left the research teams) were invited to complete a mixed‐methods cross‐sectional online survey in REDCap (Harris et al., 2009) that asked them to report which programme activities/initiatives they had engaged with and their satisfaction with their experiences. To be eligible, respondents had to have participated in at least one of the MUSQ:Impact programme activities in the prior 5 years.

### 5.1 Overall perceptions

The evaluation survey was completed by 60 people who had participated in MUSQ:Impact (Appendix Table 1). Overall, respondents had positive perceptions about the usefulness of MUSQ:Impact (Appendix Table 2). Most believed that it was useful in encouraging them to further develop their research career (95%), facilitating intellectual exchange (95%), and facilitating working collaborations (88%). The most frequently reported ways in which the programme contributed to researcher capabilities (Appendix Figure 1) included understanding translational research (52%), demonstrating research impact (33%), grant writing (31%), conducting quantitative research (31%), and engaging consumers in research (31%).

### 5.2 Webinar series

**FIGURE 2:**
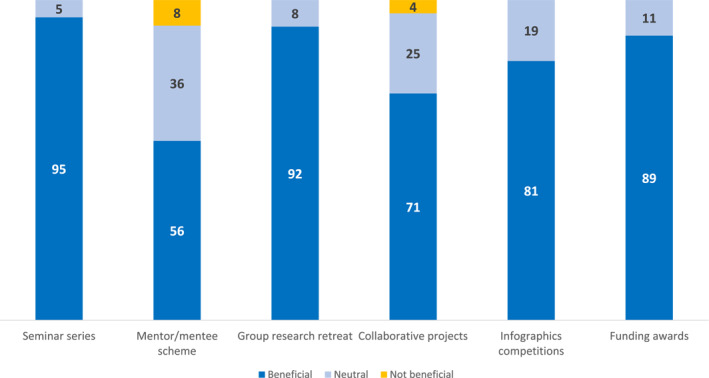
Perceptions about the value of each MUSQ:Impact Programme activity/initiative (*n* = 42 survey respondents). Data are shown as percentage.

Most survey respondents (88%) participated in the webinar series, the majority (95%) of whom found it to be valuable (Figure 2). More than half felt that the series was useful for contributing to their development as a researcher (97%; Appendix Table 2) and for contributing to their capability as a researcher (90%).

### 5.3 Mentor/mentee scheme

Around one third of respondents participated as either a mentor (31%) or a mentee (33%) in the mentoring scheme (Appendix Table 3). Reasons for not participating included logistical reasons, not being invited to participate, or not feeling the need to participate. Among those who did participate, around half (56%) of respondents believed that the scheme was beneficial, with 36% feeling neutral (Figure 2).

### 5.4 Group research retreat

Around half (55%) of respondents attended at least one of the research retreats. Overall, most (92%) found them to be beneficial (Figure 2) and believed it was very useful for intellectual exchange (90%; Appendix Table 4), collaborating and networking (86%), and enabling critical reflection and synthesis of research findings (76%).

### 5.5 Collaborative projects

Of those that attended a group research retreat, 62% participated in a collaborative group project. Overall, this was perceived to be beneficial by most (70%; Figure 2). The majority of respondents believed the projects were very useful for collaborating and networking (77%; Appendix Table 5), improving their capability as a researcher (62%), and developing their research career (69%). Project outputs have included five publications (led by EMCRs) (Egerton et al., 2022; Hall et al., 2020, 2021; Mills et al., 2019; Plinsinga et al., 2019), conference presentations, and project seed funding.

### 5.6 Infographic competition

A small number (18%) of respondents participated in the infographic competition. Reasons for not participating included not being interested or seeing the value in it, not having time, or having limited artistic ability. Among those who did participate, it was perceived by most (81%) to be beneficial (Figure 2), contributing to their capability as a researcher (71%), improving translational skills (86%) and ability to synthesise information at a lay person's level (86%), and contributing to their development as a researcher (83%) (Appendix Table 6).

### 5.7 Funding awards

Around one‐third of respondents (28%) participated in one of the funding award rounds. All (100%) believed that the award contributed to improve their capability as a researcher. Most funding awards were used for travel to conferences or to visit other researchers within the MUSQ:Impact programme, as well as for seed funding for studies.

## 6 DISCUSSION

This paper describes the structure and organisation of training and mentoring programme (MUSQ:Impact) to support musculoskeletal EMCRs. Prior to MUSQ:Impact, the senior researchers and some team members had been working together. When major collaborative group funding was obtained, it provided the capacity and impetus to initiate a formal scheme to provide their EMCRs with a breadth of experiences and opportunities to support their career development. Our evaluation survey found that the majority of MUSQ:Impact participants perceived each programme activity/initiative to be beneficial for development and capabilities as a researcher, particularly valuing the webinar series, group research retreats, and funding awards. For that reason, the senior researchers who first developed MUSQ:Impact have recognised the value in continuing the programme once the initial funding had ceased and have committed to maintaining it in the future.

Because of the geographical separation of the teams that make up MUSQ:Impact, most activities/initiatives have been organised and delivered online, allowing members from across each of the three sites on the east coast of Australia to participate. In addition, all activities and initiatives were driven and organised by EMCRs (with support and mentoring from two senior researchers), which provided them with opportunities to develop their leadership and organisational skills. Many activities (e.g. seminar series, infographic awards) have not required substantial funding or resources to organise and run and are therefore feasible for smaller research groups or those with limited funds/resources to adopt. In contrast, a challenge of some of the other activities (e.g. group research retreats and funding awards) is that they require some level of funding to be feasible.

Findings of our MUSQ:Impact evaluation highlight a number of challenges and areas for future improvements to the organisation of some activities/initiatives. The mentor/mentee scheme was perceived as being the least beneficial of all MUSQ:Impact activities. Further qualitative research is needed to explore the reasons why this was so, but might have been because of elements of its design/conduct. For example, previous research has recommended that mentee/mentoring programs should provide training for mentors and mentees (including information regarding their obligations and role in the mentee/mentor relationship) and regularly check with mentor/mentee pairs (Garringer et al., 2015; Gunn et al., 2017; Tammy D Allen et al., 2006b; Tammy D Allen et al., 2011), neither of which was included in our scheme. In the future, the scheme may be more effective and satisfactory if mentor/mentee training and check‐ins are included, and if the programme is more structured in terms of setting expectations and requirements (e.g. minimum number of mentee/mentor meetings, suggested topics for discussion). This would require a greater amount of time and effort to organise and run.

## 7 CONCLUSION

This paper presents a national training and mentoring programme that spans four musculoskeletal research teams and aims to support the career development of EMCRs. The structure and organisation of the programme, and lessons learned from its implementation, are described. This presents as a potential template for other researchers to develop such programs within their own teams and contexts.

## AUTHOR CONTRIBUTIONS

Kim L. Bennell, David J. Hunter, Paul W. Hodges, and Bill Vicenzino conceived the MUSQ:Impact programme. Belinda J. Lawford, Kim L. Bennell, and Bill Vicenzino drafted the manuscript. Rana S. Hinman, Jillian Eyles, Kim Allison, Penny Campbell, Rebecca Mellor, and Bill Vicenzino designed the survey evaluation, and collected data. All authors were members of the MUSQ:Impact Steering Committee. All revised the manuscript for intellectual content. All approved the final version for publication.

## CONFLICT OF INTEREST STATEMENT

David J. Hunter is the co‐director of the Sydney Musculoskeletal Health Flagship. In addition, David J. Hunter is the editor of the osteoarthritis section for UpToDate and co‐Editor in Chief of Osteoarthritis and Cartilage. David J. Hunter provides consulting advice on scientific advisory boards for Pfizer, Lilly, TLCBio, Novartis, Tissuegene, and Biobone.

## ETHICS STATEMENT

Ethical approval for the survey was provided by the University of Queensland Human Research Ethics Committee.

## Supporting information

Supplementary Material

## Data Availability

The data that support the findings of this study are available on request from the corresponding author. The data are not publicly available due to privacy or ethical restrictions.
