# Supplementary material for: Lessons learnt from a nationally funded training and mentoring programme for early‐mid career musculoskeletal researchers in Australia
Source: Musculoskeletal Care. 2023 Oct 16;21(4):1563–70. doi: 10.1002/msc.1820 (PMC10947136; doi:10.1002/msc.1820)
Supplement: Supplementary file 1 — Supplementary Material [file MSC-21-1563-s001.docx]

**Appendices.**

# **Appendix Table 1. Baseline characteristics of respondents to Training and Mentoring evaluation survey (N=60*).**

|  | **Mean (standard deviation) or n (%)** |
| --- | --- |
| Early-mid career researcher | 42 (70) |
| Female | 35 (76) |
| Age | 34 (9) |
| Highest qualification  Higher doctorate  Doctorate  Masters  Bachelors  Other | 3 (7)  30 (65)  8 (17)  4 (9)  1 (2) |
| Physiotherapist | 25 (54) |
| Research only position | 22 (48) |
| Teaching and research position | 9 (20) |

*Of this, only 46 responded to personal demographic questions

**Appendix Table 2. Perceived usefulness of the Training and Mentoring Program overall, n (%)**

|  | **Very useful** | **Somewhat useful** | **Neither useful or not useful** | **Not useful** |
| --- | --- | --- | --- | --- |
| Encouraging further development of research career | 22 (58) | 14 (37) | 1 (3) | 1 (3) |
| Facilitating intellectual exchange | 24 (65) | 11 (30) | 1 (3) | 1 (3) |
| Facilitating working collaborations | 20 (64) | 9 (24) | 7 (19) | 1 (3) |

**Appendix Table 3. Participant experiences with the seminar series (n=32), n (%)**

|  | **Very useful** | **Somewhat useful** | **Neither useful or not useful** | **Not useful** |
| --- | --- | --- | --- | --- |
| Contribution to development as a researcher | 20 (63) | 11 (34) | 1 (3) | 0 (0) |
| Contribution to capability as a researcher | 18 (56) | 11 (34) | 3 (9) | 0 (0) |
| Encouraging of further career development | 15 (48) | 8 (26) | 8 (26) | 0 (0) |
| Encouraging intellectual exchange | 14 (45) | 12 (42) | 4 (13) | 0 (0) |
| Facilitating collaborations | 13 (42) | 4 (13) | 12 (39) | 2 (6) |

**Appendix Table 4. Participant experiences with mentee/mentoring program, n (%) unless otherwise stated**

|  | **Mentor (n=12)** | **Mentee (n=13)** |
| --- | --- | --- |
| Number of times met (median, IQR) | 2 (103) |  |
| Happy with frequency | 7 (58) | 9 (69) |
| Would have wanted more contact | 4 (33) | 4 (31) |
| Would have wanted less contact | 1 (8) | 0 (0) |
| In terms of the mentee’s overall research/academic career aspirations/needs from the mentoring program, how did you find the mentoring program? | | |
| *Very useful* | 4 (36) |  |
| *Somewhat useful* | 3 (27) |  |
| *Neither useful nor not useful* | 1 (9) |  |
| *Not very useful* | 1 (9) |  |
| *Not at all useful* | 2 (18) |  |
| In terms of your research/academic career, how did you find the mentoring program? | | |
| *Very useful* | 4 (36) | 6 (46) |
| *Somewhat useful* | 2 (18) | 5 (38) |
| *Neither useful nor not useful* | 1 (9) | 2 (15) |
| *Not very useful* | 2 (18) | 0 (0) |
| *Not at all useful* | 2 (18) | 0 (0) |

**Appendix Table 5. Participant experiences with retreat (n=21)**

|  | **Very useful** | **Somewhat useful** | **Neither useful nor not useful** | **Not useful** |
| --- | --- | --- | --- | --- |
| Usefulness for intellectual exchange | 19 (90) | 2 (10) | 0 (0) | 0 (0) |
| Usefulness for collaboration and networking | 18 (86) | 3 (14) | 0 (0) | 0 (0) |
| To enable critical reflection and synthesis of research findings | 16 (76) | 4 (19) | 1 (5) | 0 (0) |

**Appendix Table 6. Participant experiences with collaborative projects (n=13)**

|  | **Very useful** | **Somewhat useful** | **Neither useful or not useful** | **Not useful** |
| --- | --- | --- | --- | --- |
| Usefulness for collaboration and networking | 10 (77) | 3 (23) | 0 (0) | 0 (0) |
| Usefulness for improving capability as a researcher | 8 (62) | 4 (31) | 1 (8) | 0 (0) |
| Usefulness in developing research career | 9 (69) | 3 (23) | 1 (8) | 0 (0) |

**Appendix Table 7. Participant experiences with infographic competition (n=7)**

|  | **Very useful** | **Somewhat useful** | **Neither useful or not useful** | **Not useful** |
| --- | --- | --- | --- | --- |
| Contribution to capability as a researcher | 5 (71) | 2 (29) | 0 (0) | 0 (0) |
| Contribution to improving translational skills | 6 (86) | 1 (14) | 0 (0) | 0 (0) |
| Ability to synthesise information at a lay person’s level | 6 (86) | 1 (14) | 0 (0) | 0 (0) |
| To develop digital skills | 4 (57) | 3 (43) | 0 (0) | 0 (0) |
| To enable critical reflection and synthesis of research findings | 4 (67) | 2 (33) | 0 (0) | 0 (0) |
| Contribution to development as researcher | 5 (83) | 1 (17) | 1 (17) | 0 (0) |

**Appendix Figure 1. Perceived ways in which the Training and Mentoring Program activities/initiatives contributed to capabilities as a researcher (n=42)**

Data shown as percentage
